# Male–male aggression peaks at intermediate relatedness in a social spider mite

**DOI:** 10.1002/ece3.661

**Published:** 2013-07-02

**Authors:** Yukie Sato, Martijn Egas, Maurice W Sabelis, Atsushi Mochizuki

**Affiliations:** 1 Institute for Biodiversity and Ecosystem Dynamics, University of AmsterdamP.O. Box 94240, 1090GE, Amsterdam, The Netherlands; 2 National Institute for Agro-Environmental Sciences3-1-3 Kannondai, Tsukuba, Ibaraki, 305-8604, Japan; 3 Japan Society for the Promotion of ScienceTokyo, Japan

**Keywords:** Antipredator defense, kin competition, male–male conflict, parental care, social behavior

## Abstract

Theory predicts that when individuals live in groups or colonies, male–male aggression peaks at intermediate levels of local average relatedness. Assuming that aggression is costly and directed toward nonrelatives and that competition for reproduction acts within the colony, benefits of aggressive behavior are maximized in colonies with a mix of related and unrelated competitors because aggression hurts nonkin often, thereby favoring reproduction of kin. This leads to a dome-shaped relation between male–male aggression and average relatedness. This prediction has been tested with bacteria in the laboratory, but not with organisms in the field. We study how male–male aggression varies with relatedness in the social spider mite *Stigmaeopsis miscanthi*. We sampled 25 populations across a wide geographic range between Taiwan and Japan, representing a gradient of high to low within-population relatedness. For each population the weaponry of males was measured as the length of the first pair of legs, and male–male aggression was tested by placing pairs of nonsibling males together and scoring the frequency of male death over a given period. As these two morphological and behavioral variables correlate strongly, they both reflect the intensity of male–male conflict. Our data on the social spider mite show that male–male aggression as well as weapon size strongly peak at intermediate, average relatedness, thereby confirming theoretical predictions.

Inclusive fitness theory predicts that when individuals live in groups or colonies, aggression should peak at intermediate levels of average relatedness in the colony. Here, we study how male–male aggression varies with average relatedness in naturally occurring colonies of the social spider mite *Stigmaeopsis miscanthi*. In support of theory, male–male aggression and weapon size strongly peak at intermediate average relatedness.

## Introduction

Kin selection provides an explanation of male–male aggression (Reinhold 10; Gardner and West 2). A classic example is given by fig wasps, showing interspecific variation in male–male aggression (Hamilton 5). Within the figs where they develop, the wingless males search for available females to mate with and they compete with their rivals (i.e., local mate competition where the mating group comprises of all wasps within one fig). After mating, the winged females disperse for oviposition and the wingless males die (Hamilton 5). Species with wingless males expressing various forms of highly modified fighter morphs exhibit less female-biased sex ratios. According to local mate competition theory (Hamilton 4) female-biased sex allocation is adaptive for mothers when their sons compete for mating partners among themselves rather than with other males. Hence, the observation on different sex ratios may indicate lower average relatedness in mating groups of species with fighter morphs than in those of species without fighter morphs (Hamilton 5). However, in an analysis controlling for the phylogenetic relationships among fig wasps, West et al. (33) did not find that male–male aggression declined with the average genetic relatedness expected from the sex ratio. Instead, they found that male–male aggression negatively correlated with female density. This empirical result prompted the question how competition between kin affects the evolution of male–male lethal fight, especially in “viscous populations.” The answer came from two models, one by Reinhold (10) and another by Gardner and West (2). Both models predict that the relation between male lethal fight and average genetic relatedness is not linear and not negative, but dome shaped: male–male aggression is expected to peak at intermediate relatedness. The key assumptions underlying this prediction are that (1) there are local mating groups of males in a population that compete for females as mating partners and this competition is manifested by some form of aggression among males, and (2) males have the ability to discriminate between kin and nonkin males and use this ability to decide on male killing actions they will manifest toward their rival. At some intermediate level of average relatedness in a group, some degree of male killing behavior would be favored because males meet males varying in genetic relatedness, and some of them are more related to the actor than others. If the average relatedness in a group is very high, however, male killing behavior would not be favored because the males meet only kin, and if average relatedness in a group is very low, male killing behavior would not be favored either because males meet only nonkin.

**Figure 1:**
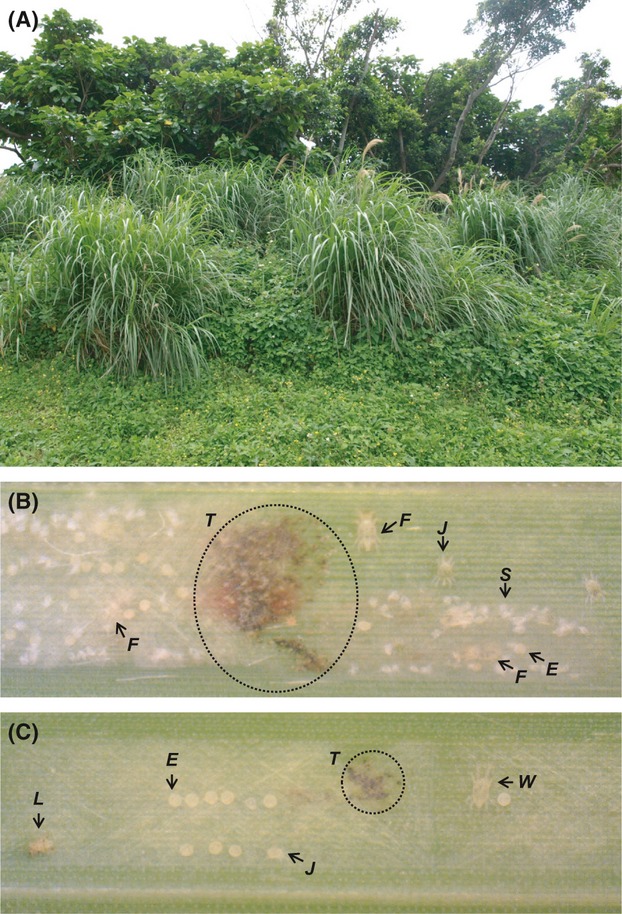
Photos of (A) a stand of Chinese silver grass, the host plant of the social spider mite *Stigmaeopsis miscanthi*, (B) a nest of *S. miscanthi* on a leaf of Chinese silver grass viewed from above, with eggs (E), juveniles (J), shed skins of molting stages of the mite (S), three adult females (F), and a pile of feces (“toilet”; T), covered by webbing (gray haze in the photo), and (C) a winner (alive male at the right hand; W) and a loser (dead male at the left hand; L) of male–male lethal fight in a nest of *S. miscanthi*.

Here, we study the relation between aggression and relatedness, using the herbivorous, social spider mite *Stigmaeopsis miscanthi* (Saito). This mite forms colonies on Chinese silver grass (Saito et al. 25; Fig. A), each colony consists of a silken web on the undersurface of grass leaves (Fig. 1B), and each web serves as a nest for groups of mites that differ in kinship depending on the level of inbreeding in subsequent generations. This mite species often shows parental care in that males cooperate by defending the offspring they fathered by counterattacking those predatory mites that intruded the nest (Saito 12,b; Yano et al. 34). This counterattack is not aimed at the adult females of the predatory mite, but rather at their larvae. As predatory mites are synovigenic, they need prey to lay eggs and they will not eat all prey in a nest because the remainder is required as food for the development of the predator's offspring. By the time the adult females of the predatory mites have left the nest, nest mates of the spider mite including males cooperate in counterattacking the juveniles of the predatory mites, thereby defending what is left of their offspring. This behavior represents one of the main reasons why this mite is considered to be a social spider mite (Saito 21) and leads us to suspect an ability to discriminate kin from nonkin in this species. We are aware of one study that tested for this ability but failed to find evidence (Saito 17). This study simply observed male fights between two males from a single unfertilized mother and between two males from different subcultures. If the males simultaneously confront both kin and nonkin males in a nest, they possibly change their behavior (Saito 17). In agreement with Saito (17), we consider this absence of evidence, not evidence for absence of kin discrimination ability. In fact, in the spider mite species *Tetranychus urticae* there is solid evidence for strain-specific assortative female–male mating (Vala et al. 32) as well as for kin discrimination in mate choice (Tien et al. 31), which arguably makes the hypothesis on kin discrimination ability in the social spider mite *S. miscanthi* plausible.

Males do not only show aggression toward predators but also toward conspecific males (Saito 15). In their competition for females as mating partners, they kill rival males inside nests, and thereby establish their own harem (Fig. 1C). Male–male aggression (quantified as the probability of lethal combat) is highly variable among populations, and it is negatively correlated with winter harshness (Saito 19). This negative correlation with male–male aggression can be regarded as evidence for kin selection, given that winter harshness is an indicator of genetic relatedness in this mite species (Saito 19). Females enter diapause to overwinter (Saito et al. 24), but males cannot enter diapause and their chance to survive the winter largely depends on winter harshness: Males survive better in regions with mild instead of severe winters (Saito 19). In spring, the females that overwintered successfully and emerged from their winter refuge are usually isolated and distant from each other and they individually create webs of silk on leaves and start to lay eggs in the silken nests. Like all spider mites, the mite species considered here has regular, haplodiploid sex determination and reproduce by arrhenotokous parthenogenesis. Thus, if nearby there are no males that survived the winter, overwintering females that are noninseminated (~10–20% of overwintering females; Saito 14) are forced to produce haploid sons (by arrhenotokous parthenogenesis) and by mother–son mating they can produce diploid daughters and next generations (Saito 14). The females that are inseminated before winter can produce diploid daughters without mother–son mating, but their daughters are likely to sibmate with their sons. However, if there are males nearby that survived the winter, the females can establish their colonies without mother–son mating and without daughter–son mating regardless of the female insemination status before winter. Mother–son mating at the nest foundation can create considerable differences in the average relatedness between fathers and sons and among sons (Appendix S1). Thus, severe winter harshness can generate inbreeding, thereby increasing the genetic relatedness among nest members, whereas mild winters create low relatedness in the nests.

**Figure 2:**
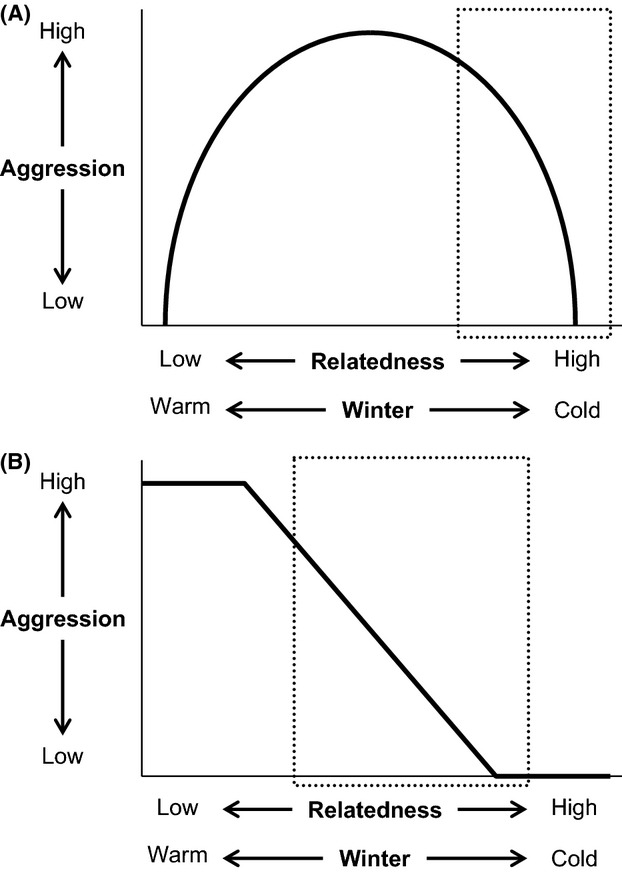
Schematic relationship between male–male aggression and genetic relatedness expected by (A) models involving kin selection and kin competition (Reinhold 10; Gardner and West 2), and (B) the model involving a trade-off between male–male aggression and cooperation emerging from the genetic relatedness (Saito 20; Saito and Mori 22). The dotted line squares indicate the estimated range observed in the previous studies using *Stigmaeopsis miscanthi* (Saito 19; Saito and Sahara 23).

**Figure 3:**
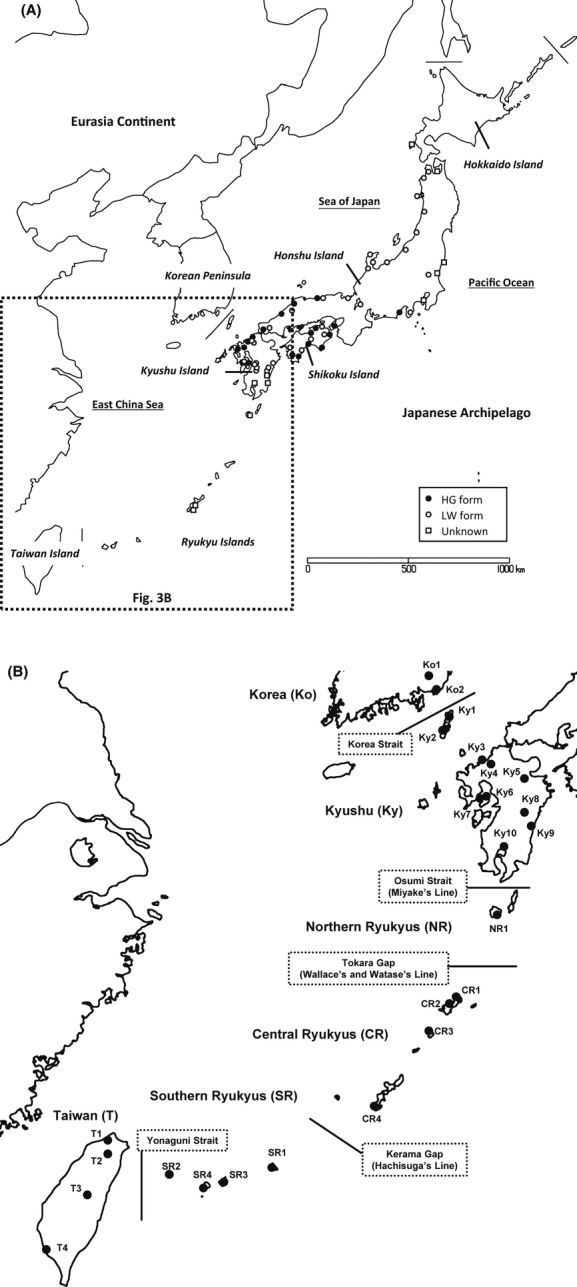
Locations of *Stigmaeopsis miscanthi* populations in a study prior to ours (Saito and Sahara 23; A) and the research area in the study described in this article (B). (A): Filled and open circles indicate high- and low-aggression form populations, respectively. Open squares indicate populations in which aggression has not yet been observed. (B): Filled circles indicate the locations of *S. miscanthi* used in this study. These populations are grouped into six regions based on current faunal characteristics and geographic features and are numbered in order of north latitude in each region.

In this article, we use *S. miscanthi* populations to test whether male–male aggression peaks at intermediate levels of average (within-colony) relatedness (Fig. 2A; Reinhold 10; Gardner and West 2). In this mite, males are part of local mating groups and they are likely to compete for females. This includes the case where there are only related males because (1) females produce usually more than one male per nest (despite the strong female bias in sex ratio), (2) males spend most of their lives inside nests of woven silk (Saito 11, 18; Sato et al. 30), (3) they rarely leave these nests as the silken web serves to protect them against predators, rain, and wind (Mori and Saito 9), and because (4) the silken web harbors their offspring which they can help to protect against predators (Saito 12,b; Yano et al. 34). Thus, the key assumptions of the models of Reinhold (10) and Gardner and West (2) are fulfilled in that there is direct evidence for the existence of local mating groups and between-group variation in genetic relatedness and the indirect (circumstantial) evidence for kin recognition ability, as explained above. Previous studies to assess the relation between relatedness and aggression experimentally (Saito 19; Saito and Sahara 23) were limited in that the populations under study had a narrow range of relatively high relatedness (the right part in Fig. 2A) (Sato et al. 28,b; Sato and Saito 27). Because winter harshness declines toward the south, we focused on populations to the south of these previous studies. Going from northern to southern populations we expect male–male aggression to reach a peak and then decline (Fig. A). The alternative hypothesis is that male–male aggression continues to increase toward the south, as predicted by Saito (20) and Saito and Mori (22) (Fig. 2B). Hence, we investigated male–male aggression in *S. miscanthi* in populations from the Korean Peninsula to Taiwan Island and from the southern part of Japan as studied by Saito (19) and Saito and Sahara (23) (Fig. 3A). We estimated winter harshness at the mite collection sites by using location-specific climatic data, and assessed the relation between male–male aggression and the estimated winter harshness. Because male aggressive behavior may easily vary depending on experimental conditions, we also quantified a morphological (and hence nonbehavioral) correlate of male aggression: males with longer first legs usually win in male–male combat (Saito 16). This mite species consists of two forms in the mainland of Japan, which differ in male–male aggression: the high-aggression form and the low-aggression form (Saito and Sahara 23). Subtle genetic differences between the forms (Ito and Fukuda 8; Sakagami et al. 26) and an incomplete reproductive barrier between them (Sato et al. 28,b) were found. However, we analyzed the molecular phylogenetic relation among the populations that we used in this study (the outgroup is its closely related species, *S. longus*; Y. Sato et al. unpubl. data). The result indicate that it is plausible to consider the populations we investigated in this study as closely related and belonging to one and the same species. Hence, we analyzed these populations without consideration of forms and their phylogenetic relations.

## Methods

### Host plant and mite collection

The Chinese silver grass *Miscanthus sinensis* Andersson was collected from Tsukuba (Ibaraki Prefecture, Japan) in March 2009. Its roots were pulled apart and the resulting clones were grown in a greenhouse to be used for mite rearing and experiments. *Stigmaeopsis miscanthi* was collected from 25 sites: the Korean Peninsula (two sites), the Kyushu region (10 sites), the Ryukyu Islands (nine sites including one site in the Northern Ryukyus, four sites in the Central Ryukyus, and four sites in the Southern Ryukyus), and the Taiwan Island (four sites; Fig. 2B). Mite collection was conducted from December 2008 to July 2009. Collected mites were reared on host plant leaves under controlled conditions at 18–25°C, 60–80% relative humidity (RH), and 15:9 h light:dark (L:D), and were maintained at 25 ± 1°C for at least 1 month before the experiments. Note that two forms of *S. miscanthi* have been described before: the HG form only from Southwest Japan and the LW form occurring throughout Japan, excluding Hokkaido Island (Fig. 3A; Saito and Sahara 23).

### Male–male aggression

A piece of detached *M. sinensis* leaf (2.0 × 0.5 cm) was placed on water-soaked cotton wool and the cut edges were covered by thin strips of wet cotton wool. Two females, selected from the same culture as the males, were placed in the leaf arena and allowed to construct a nest and deposit several eggs for 4–5 days. The females were removed from the arena, and two nonsibling males were then placed there. The nonsibling status of these males was ensured by taking one male from each of two subunits that were created from the rearing 1 month before the experiment. The survival of the males was observed daily under a dissecting microscope for 5 days. This treatment was conducted 29–35 times in each population. Male–male aggression was evaluated in terms of the probability of one of the two males dying within the period of 5 days. The experiments were conducted at 25 ± 1°C, 60–80% RH, and 15L:9D.

### Estimation of winter harshness

The mean daily minimum temperature from December to February (MDMT-Winter), from 1971 (or 1979 depending on the weather station) to 2000, and latitude and altitude of 117 weather stations located in the Kyushu region (Fukuoka, Kagoshima, Kumamoto, Miyazaki, Nagasaki, Oita, and Saga Prefectures) and Okinawa Prefecture were obtained from AMeDAS climatic data supplied by the Japan Meteorological Agency (latitude range: 24°03.3′–34°11.8′N; altitude range: 2–1142.3 m). Using these data, we constructed a general linear model with MDMT-Winter as the response variable and north latitude and altitude as the explanatory variables. This general linear model was used to estimate MDMT-Winter at each mite collection site. This analysis was performed with the freeware statistical package R version 2.11 (R Project for Statistical Computing, http://www.r-project.org).

### Male weapon morph

From each leaf culture, 16–22 males were selected arbitrarily. These males were prepared separately as slide specimens using Hoyer's solution. The specimens were dried by pressing them with a 10-g weight at 45°C for 3 days to flatten the body for accurate measurement. The lengths of the tibia, tarsus, genu, and femur on legs I and III were measured using a photomicrograph and image-processing software (Image J ver. 1.41; National Institutes of Health, Bethesda, MD). The relative length of leg I to leg III (leg I/leg III) was calculated from the totals of the four leg segment lengths. The relative lengths of male leg I (leg I/leg III) were log transformed, and the population average value of the transformed data was used as the male weapon morph of the population.

### Data analysis

Three generalized linear models with quasibinomial error distribution were constructed, in which the response variable was male–male aggression (Models 1, 2, and 3). In Model 1, the explanatory variable was the male weapon morph to test a linear relation between male–male aggression and male weapon morph. In Model 2, the explanatory variable was winter harshness to test linear relation between male–male aggression and winter harshness. In Model 3, to test a dome-shaped relation between male–male aggression and winter harshness, the explanatory variables were winter harshness and winter harshness squared. Two general linear models (Models 4 and 5) were constructed with male weapon morph as the response variable and the following explanatory variables: winter harshness in Model 4 and winter harshness and winter harshness squared in Model 5. To test linear and dome-shaped relations of either male aggression (Models 2 and 3) or male weapon morph (Models 4 and 5) with winter harshness, a likelihood ratio test was applied. The data analyses were performed using the statistical package R, version 2.14.1 (R Project for Statistical Computing, http://www.r-project.org).

## Results

### Estimation of winter harshness

The function used to estimate the MDMT-Winter of each mite collection site, which was obtained using climatic and location data from 117 weather stations, was as follows: MDMT-Winter = −1.747 × NL − 0.008 × AT + 60.289, where NL is north latitude and AT is altitude. This function explained climatic and location data well (*R*^2^ = 0.912, *F*_2,114_ = 599.5, *P *<* *0.001), and the effects of both explanatory variables were significant (North latitude: *t *=* *−31.778, *P *<* *0.001; Altitude: *t *=* *−8.569, *P *<* *0.001).

### Male–male aggression, winter harshness, and male weapon morph

**Figure 4:**
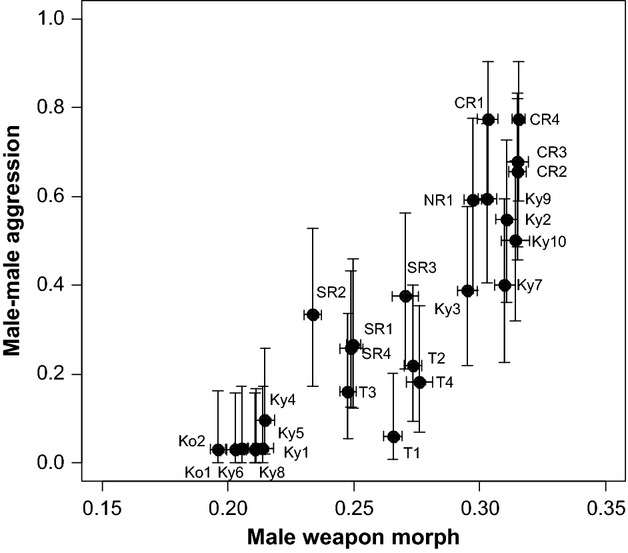
Relation between average male–male aggression per population (each based on at least 30 replicates; see M&M) and average relative length of male leg I, as a measure of male investment in weaponry. Vertical and horizontal error bars indicate 95% CI and SEM, respectively. For population locations, see Figure 3B.

**Figure 5:**
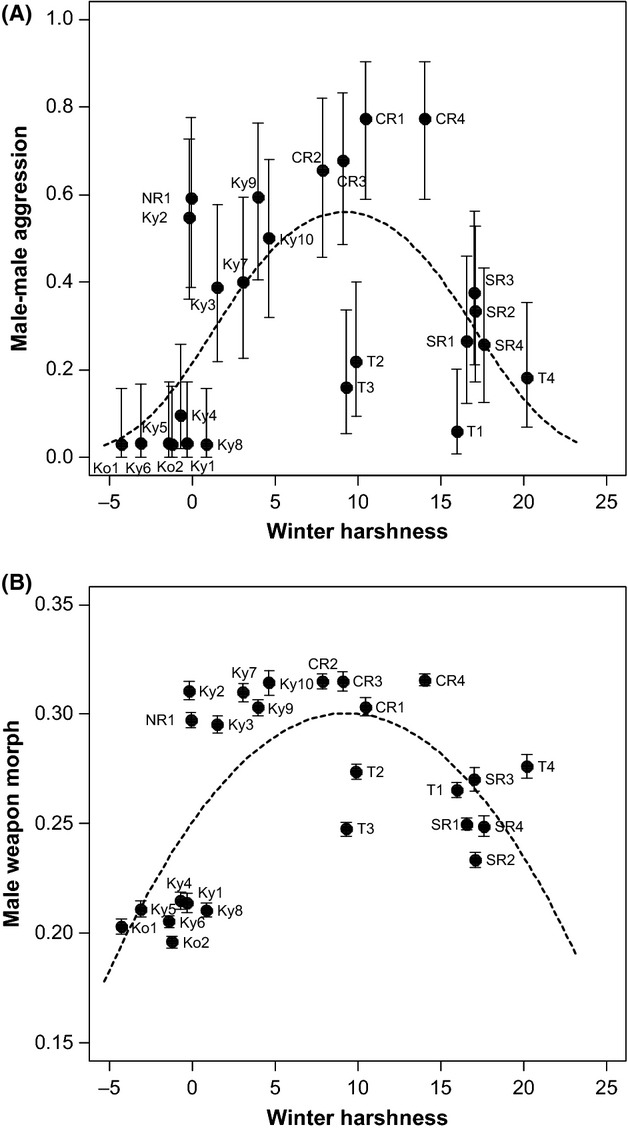
Relations between (A) average male–male aggression and winter harshness and between (B) average male weapon morph and winter harshness. (A): Error bars indicate 95% CI. The dotted line illustrates the nonlinear relation supported by the statistical analysis (Model 3 in Table 1). (B): Error bars indicate SEM. The dotted line illustrates the nonlinear relation supported by the statistical analysis (Model 5 in Table 1). For population locations, see Figure 3B.

Male–male aggression varied greatly among the populations (Figs. 4, 5). The male weapon morph explained the male–male aggression well (Model 1 in Table 1; Fig. 4). The winter harshness explained the male–male aggression and the male weapon morph well too (Models 3 and 5 in Table 1; Fig. 5). Model 3 testing the dome-shaped relation of male–male aggression with winter harshness fitted much better than Model 2 testing a linear relation with winter harshness (Likelihood ratio test: df = 1, *χ*^2^ = 84.96, *P *<* *0.001; Fig. 5A). Model 5 testing the dome-shaped relation of male weapon morph with winter harshness fitted much better than Model 4 testing a linear relation with winter harshness (Likelihood ratio test: df = 1, *F *=* *14.023, *P *<* *0.01; Fig. 5B).

**Table 1:** Results of model 1: a quasi-binomial generalized linear model to test a linear relation between male–male aggression and male weapon morph, model 3: a quasi-binomial generalized linear model to test a dome–shaped relation between male–male aggression and winter harshness, and model 5: a general linear model to test a dome-shaped relation between male weapon morph and winter harshness

Predictor	Coefficient	SE	*t*	*P*
Model 1				
(Intercept)	−9.335	1.130	−8.264	<0.001
Male weapon morph	31.317	3.963	7.902	<0.001
Model 3				
(Intercept)	−1.295	0.344	−3.763	<0.01
Winter harshness	0.333	0.097	3.455	<0.01
Winter harshness^2^	−0.018	0.006	−3.285	<0.01
Model 5				
(Intercept)	0.251	0.0089	28.088	<0.001
Winter harshness	0.011	0.0026	4.192	<0.001
Winter harshness^2^	−0.001	0.0002	−3.745	<0.01

## Discussion

We showed that the relation between male–male aggression in a social spider mite and the estimated winter harshness is dome-shaped rather than linear (Fig. 5). Assuming winter harshness is a proxy of average relatedness, this result is in agreement with the predictions from game theoretical models that take both kin selection and local kin competition into account (Fig. 2A; Reinhold 10; Gardner and West 2). In addition, we found that male–male aggression is strongly related to the morphology of the male fighter morph (Model 1 in Table 1; Fig. 4), thereby increasing confidence in our assessments of male–male aggression.

Evidence for the dome-shaped relation predicted by game theoretical models has been obtained for laboratory populations of bacteria-producing bacteriocins that kill nonkin cells only (i.e., perfect kin recognition) and effectively commit suicide to free up extra resources for their kin (i.e., the aggressive behavior is clearly harmful to the actor) (Gardner et al. 3; Inglis et al. 6, 7). However, to the best of our knowledge, our findings provide first evidence from natural populations along a geographic cline, supporting the dome-shaped relation predicted. They do not contradict previous studies (Saito 19; Saito and Sahara 23) in which a negative correlation was found between male–male aggression and the expected relatedness in the same spider mite species because the range of average relatedness levels in the populations used in these studies was narrow and biased to relatively high relatedness (as illustrated in Fig. 2A).

The dome-shaped relation we found cannot emerge solely from differences in average relatedness. It can emerge when additionally local competition between males for mating opportunities is taken into account (Reinhold 10; Gardner and West 2). Otherwise, a linear relation would be expected, as argued by Saito and Mori (22). According to Saito (20) and Saito and Mori (22), nest defense by two males is far more effective than that by one male (Saito 13; Yano et al. 34). Thus, the killing of conspecific males within nests has two contrasting effects: it decreases fitness by exposing mating partners and offspring to the risk of predation, but it increases the fitness of an individual male by monopolizing females (Saito 15). If male killing has only the latter effect (as in fig wasps), there is no gain in giving up mating chances to promote those of other males (Saito 20; Saito and Mori 22), but if male killing has the former effect, male–male cooperation may promote inclusive fitness and this counteracts the selfish act of male killing. Thus, there is a trade-off between male–male aggression and male–male cooperation, and the most favorable combination depends on the genetic relatedness between males and between males and nest members (Saito 20; Saito and Mori 22). This trade-off may explain why there is an effect of relatedness on aggressive behavior in the spider mite but not in the fig wasps: Within the fig there are no enemies that fig wasp males can cooperatively counterattack. In fact, there is only one report on frugivorous insects that as a by-product kill the fig wasps inside the fig (Bronstein 1). These “enemies” are too big and therefore unlikely to suffer from counterattack by fig wasp males, even when they would do this cooperatively. Thus, following Gardner and West (2) to understand the dome-shaped relation in the social spider mite, there is a need to test whether male killing represents spiteful behavior by measuring the fitness benefits and costs of male lethal fight to the actor as well as to the recipient.
