# Supplementary material for: Male–male aggression peaks at intermediate relatedness in a social spider mite
Source: Ecol Evol. 2013 Jul 2;3(8):2661–9. doi: 10.1002/ece3.661 (PMC3930045; doi:10.1002/ece3.661)
Supplement: Supplementary file 1 [file ece30003-2661-SD1.docx]

Appendix 1. Mother-son mating at foundation of nests in the spring affects the relatedness between fathers and sons and between sons.

*Stigmaeopsis miscanthi* is a social spider mite, and the adult and immature mites live gregariously in woven nests with two to three generations overlapping in each nest (Saito 1997). Hence, male fight is expected mainly to occur between fathers and sons and between sons and therefore we calculate average relatedness for both these categories. Just like other spider mites, the mite is haplo-diploid: females develop from fertilized eggs (2n) and males from unfertilized eggs (n). We label the two alleles of the mother on an arbitrary locus as *A* and *B* and that of the father as *C*. In Fig. A1 we illustrate the different genotypes per generation, as well as the frequency of these genotypes, that may result from inbreeding or outbreeding in the first generation followed by several generations of inbreeding. Under first-generation inbreeding the mother (*AB*) deposits unfertilized eggs and mates with her son (assumed, without loss of generality, to have genotype *A*) who developed from one of the unfertilized eggs in the same nest. This is followed by brother-sister mating for several generations. Under first-generation outbreeding the mother (*AB*) mates with an unrelated father (*C*) to establish a nest in spring, and this is also followed by brother-sister mating for several generations (Fig. A1). Note that the fathers in this haplo-diploid reproduction system do not have sons, but do pass on genes to the grandsons via daughters, and may be related to the sons of their mating partners if sib-mating occurs.

To calculate average relatedness between individuals from the expected average frequency of alleles shared through recent ancestry, we assume that the genome contains very many unlinked loci. Under first-generation inbreeding, the average relatedness in the first generation between father and sons is 0.5 (Fig. A2a) and that among sons is also 0.5 (Fig. A2b). After a small increase in the second generation, each of the two types of average relatedness remains around 0.5. Under first-generation outbreeding, the average relatedness in the first generation between father and sons is 0 (Fig. A2a) and that among sons is 0.5 (Fig. A2b). After the second generation, the average relatedness between father and sons increases gradually, but that among sons decreases slightly (Fig. A2a,b). Neither of them reaches the level observed under first-generation inbreeding (Fig. A2a,b).
